# APOE ε4 genotype confers risk for Alzheimer Disease in Cuban Americans

**DOI:** 10.1002/alz.092880

**Published:** 2025-01-09

**Authors:** Jose Javier Sanchez, Bilcag Akgun, Kara L. Hamilton‐Nelson, Joe Rivero, Katrina Celis, Larry D. Adams, Glenies S Valladares, Concepcion Silva‐Vergara, Vanessa C Rodriguez, Pedro R. Mena, Patrice L. Whitehead, Michael B. Prough, Anthony J. Griswold, Clifton L. Dalgard, Katalina F McInerney, Jeffery M. Vance, Michael L. Cuccaro, Gary Beecham, Farid Rajabli, Margaret A Pericak‐Vance

**Affiliations:** ^1^ John P. Hussman Institute for Human Genomics, University of Miami Miller School of Medicine, Miami, FL USA; ^2^ John P. Hussman Institute for Human Genomics, University of Miami Miller School of Medicine, Miami, FL, USA, Miami, FL USA; ^3^ Dr. John T. Macdonald Foundation Department of Human Genetics, University of Miami Miller School of Medicine, Miami, FL USA; ^4^ Department of Anatomy, Physiology, and Genetics, Uniformed Services University of the Health Sciences, Bethesda, MD USA; ^5^ University of Miami Miller School of Medicine, Miami, FL USA; ^6^ Department of Neurology, University of Miami Miller School of Medicine, Miami, FL USA; ^7^ 1501 NW 10th Avenue, Miami, FL USA

## Abstract

**Background:**

The risk of Alzheimer Disease (AD) conferred by APOE and other genetic factors differ across populations. The Cuban American (CA) population is 3‐way admixed (European, African, and Amerindian) and vastly underrepresented in genetic studies. Previous genetic studies in this population have solely focused on APOE in AD and shown conflicting results regarding its effects. We aimed to evaluate the effect of the APOE genotype and other variants in potential causative AD genes in the CA population.

**Methods:**

The CA dataset includes Whole Genome Sequencing (WGS) and Array data from 141 individuals (40 AD, 50 mild cognitive impairment [MCI], 51 cognitively unimpaired) ascertained through the Cuban American Alzheimer’s Disease Initiative (CuADI). To assess the effect of APOE‐ε4 allele dosage on AD, we used a generalized estimating equations (GEE) method adjusting for sex, age, and population substructure. Subsequently, we extracted and evaluated all variants in 7 potential causative genes: APOE, APP, MAPT, PSEN1, PSEN2, SORL1, and TREM2. We calculated the admixture proportion using a model‐based clustering algorithm implemented in the ADMIXTURE software.

**Results:**

The APOE‐ε4 alleles were significantly associated with AD risk (p=0.013; OR=3.4[1.3‐8.7]). The causative genes analysis showed a known exonic variant (rs117260922; p.E270K) in the SORL1 gene in 1 AD and 2 MCI individuals, and a novel splicing variant (c.308‐1G>A) in the MAPT gene in an AD individual. Admixture analysis revealed average proportions of 83.4% European, 12.4% African, and 4.3% Amerindian in the CA cohort.

**Conclusions:**

We showed that the APOE‐ε4 genotype conferred a risk for AD in the CA population. The effect size of the APOE‐ε4 in our study was found to be similar to a recent study in large non‐Hispanic white (NHW) cohort (OR=3.32, Kunkle et al. 2019). High European background in the CA population is likely cause to this similarity. Our results also support that the E270K variant in the SORL1 gene, previously linked to AD in Caribbean Hispanics and NHW studies, contributes to the risk of AD. These findings provide new insights into the genetic architecture of AD in the CA population.

